# Integrating emotional vocabulary in EFL education: a model for enhancing emotional intelligence in pre-service EFL teachers

**DOI:** 10.3389/fpsyg.2024.1508083

**Published:** 2025-01-27

**Authors:** Olusiji Lasekan, Margot Godoy, Claudia Méndez-Alarcón

**Affiliations:** ^1^Departamento de Educación e Innovación, Universidad Católica de Temuco, Temuco, Chile; ^2^Languages Coordination, DITFO, Universidad de La Frontera, Temuco, Chile; ^3^Facultad de Ciencias Humanas, Universidad Autónoma de Baja California, Mexicali, Mexico

**Keywords:** emotional intelligence, emotional vocabulary, pre-service EFL teachers, systematic teaching model, socio-emotional learning

## Abstract

This study investigates the role of emotional vocabulary in fostering emotional intelligence (EI) among pre-service EFL teachers, focusing on the Headway series. The general objective was to quantify, categorize, and classify emotion vocabulary across proficiency levels and to develop a systematic teaching model to enhance EI in pre-service teachers. A mixed-methods approach was employed: quantitative analysis identified and categorized emotional vocabulary using established lexicons, and qualitative thematic analysis aligned this vocabulary with key EI components such as self-awareness, emotional regulation, and empathy. To develop the systematic teaching model, a design-based research methodology was utilized, incorporating iterative refinement based on empirical findings and expert feedback. Grounded in Goleman’s Emotional Intelligence Theory, the study revealed a progressive increase in emotional vocabulary, with more complex emotions introduced at advanced levels. Positive emotions were more frequent at beginner levels, while negative and neutral emotions increased at higher stages, supporting self-regulation and empathy development. The systematic teaching model proposed in the study addresses key EI components such as self-awareness, social skills, and emotional regulation by scaffolding emotional vocabulary instruction across proficiency levels. This model provides a structured framework to equip educators with the emotional and linguistic tools necessary for effective classroom management and enhanced student outcomes. This research holds pedagogical implications for teacher training programs, recommending that emotional vocabulary and EI be integrated into pre-service education to improve classroom management and student outcomes.

## Introduction

Socio-emotional development in higher education and teacher training is essential for effective teaching and learning, encompassing emotional intelligence (EI), social skills, and stress management. Integrating socio-emotional learning (SEL) into curricula helps educators address classroom challenges and foster inclusive environments. Teachers with strong socio-emotional skills improve classroom dynamics, job satisfaction, and student outcomes, including mental health and academic success (Molina-Moreno et al., 2024; Shankland et al., 2024). A holistic approach combining emotional and cognitive competencies enhances student well-being and future success (IndellIcato, 2024). Implementation requires aligning practices with theories like Goleman’s, focusing on empathy and stress management, and using robust evaluation models (Calderón Calderón, 2024). Resistance to change in traditional models highlights the need for a pedagogical shift to integrate emotional and cognitive processes (IndellIcato, 2024). Despite challenges, SEL offers transformative opportunities for education (Shankland et al., 2024).

The significance of emotion vocabulary in EFL (English as a Foreign Language) textbooks has gained recognition in recent years, as it plays a crucial role in enabling learners to communicate effectively and develop sociopragmatic competence, which is essential for navigating social interactions. Despite this, emotion vocabulary remains underrepresented in many EFL textbooks, leaving learners ill-prepared to express emotions in a foreign language (Dewaele, 2015). Some of the progress highlighted include improvements in the incorporation of emotion vocabulary in certain textbooks (Sánchez and Pérez-García, 2020), yet a significant gap persists. This deficiency is concerning, as research shows that exposure to emotion-laden words and texts can positively influence vocabulary acquisition and retention, with neutral and negative emotion-laden texts predicting better learning outcomes than positive texts (Driver, 2022). Furthermore, teachers themselves often have a limited emotional vocabulary, which can hinder their ability to support students’ development of a robust emotional lexicon (Alzina and Guiu, 2018). Addressing the emotional vocabulary gap in EFL textbooks and instruction is essential for enhancing learners’ language skills and helping them use language in socially and emotionally appropriate ways.

Emotional intelligence (EI) plays a crucial role in enhancing language learning, particularly for pre-service EFL teachers, as it significantly contributes to teacher effectiveness, student achievement, and classroom management (Coombe et al., 2020). Research shows a positive relationship between teachers’ EI and students’ language achievement (Al Jaberi et al., 2024), and EI can be developed through reflective practices such as journaling and classroom discussions, which help teachers build self-awareness and emotional regulation (Zadorozhna et al., 2018). For pre-service teachers, EI awareness impacts their teaching experiences and efficacy (Dick-Bursztyn, 2023; Koçoğlu, 2011). Incorporating EI training in EFL education fosters emotional awareness and communication skills, enhancing students’ language competence (Balasubramanian and Al-Mahrooqi, 2016). Furthermore, explicit instruction in emotion vocabulary plays a vital role in improving pre-service teachers’ emotional awareness and communication abilities, as emotion-laden texts have been shown to aid in vocabulary retention and comprehension, especially when neutral or negative emotional contexts are involved (Driver, 2022). Therefore, integrating EI instruction in EFL teacher training programs is essential to promote emotional awareness, improve teaching effectiveness, and support classroom dynamics (Alrefaai and Shah, 2020).

Fostering emotional intelligence (EI) in pre-service Chilean EFL teachers is increasingly recognized as a critical element for effective teaching, teacher well-being, and classroom management. EI, which encompasses the ability to recognize, understand, and manage one’s emotions and the emotions of others, is essential for teachers to navigate the emotional complexities of the classroom and maintain high levels of job performance and satisfaction (Extremera and Fernández-Berrocal, 2004). In Chile, as in many other countries, teachers face stressful environments, making EI crucial for preventing burnout and promoting resilience. Research indicates that teachers with higher EI levels are more effective in managing stress, improving student outcomes, and fostering positive classroom environments (Vesely-Maillefer and Saklofske, 2018). However, despite its importance, EI is often underdeveloped in Chilean pre-service teacher training programs, limiting the ability of new educators to manage their emotional well-being and engage effectively with students (Valenzuela-Zambrano et al., 2021). Many Chilean teachers perceive themselves as having low levels of EI, which can hinder their ability to manage classroom dynamics and cope with the emotional demands of the profession (Valenzuela-Zambrano et al., 2021). This self-perception is linked to the inadequate integration of EI training in teacher education programs (Muñoz, 2022). Without comprehensive EI training, pre-service teachers enter the workforce without the emotional tools needed to manage stress, avoid burnout, and foster positive relationships with students and colleagues. This lack of emotional preparedness contributes to lower job satisfaction, increased teacher turnover, and diminished classroom effectiveness (Delgado-Bello et al., 2021).

While the significance of emotional vocabulary in EFL education is increasingly acknowledged, prior research has several limitations that hinder its practical application. For instance, studies often focus on the presence of emotional vocabulary in textbooks without critically analyzing its pedagogical implications or its alignment with emotional intelligence development (Kang, 2022; Thuy, 2024). Additionally, there is a lack of exploration into how cultural contexts influence the selection and teaching of emotional vocabulary. Research has shown that cultural differences significantly impact emotional expression, with some cultures emphasizing collectivist emotional norms, such as empathy and harmony (Kikutani et al., 2024), while others prioritize individual emotional expression and independence (Imada, 2012). These cultural nuances play a vital role in shaping how emotional vocabulary is represented in teaching materials, potentially limiting their relevance in diverse educational contexts. Addressing these gaps, this study not only examines the presence and categorization of emotional vocabulary in EFL textbooks but also considers how cultural factors influence its pedagogical application, providing a more comprehensive framework for fostering emotional intelligence in pre-service teachers.

Despite growing recognition of the importance of emotional intelligence (EI) and emotion vocabulary in language learning, existing EFL (English as a Foreign Language) materials, including popular textbooks like the Headway series, which is widely used to train pre-service EFL teachers in Chile, often lack a systematic focus on integrating emotional intelligence training and emotion vocabulary development. The underrepresentation of emotion vocabulary leaves learners ill-equipped to communicate emotions effectively in a foreign language, impacting their sociopragmatic competence and emotional awareness. Moreover, pre-service EFL teachers are not adequately prepared to teach emotional vocabulary, which could foster emotional intelligence in their future classrooms. This lack of emphasis on emotional intelligence and emotion vocabulary in current EFL materials necessitates a closer investigation to bridge this gap.

Thus, this study seeks to address the critical gap in the integration of emotional vocabulary in EFL materials and its potential to foster emotional intelligence (EI) in pre-service teachers. Specifically, it aims to quantify, categorize, and classify emotion vocabulary in the widely used Headway series and develop a systematic teaching model to enhance socio-emotional competencies in EFL teacher training. The following research questions guide this investigation:

1. How many emotion vocabulary words are present across all levels of the Headway series?
2. How can emotion vocabulary in the Headway series be categorized according to basic emotions (joy, anger, fear, sadness, surprise, and disgust)?
3. How can emotion vocabulary in the Headway series be classified by valence (positive, negative, neutral)?
4. How is emotion vocabulary in the Headway series distributed by word class (verb, noun, adjective, adverb)?
5. How can the findings on emotion vocabulary be used to develop a systematic teaching model for fostering emotional intelligence in pre-service EFL teachers?

By addressing these questions, the study aims to build a foundation for the systematic inclusion of emotional vocabulary in EFL teacher training and explore its pedagogical implications for fostering EI. The objectives are to provide a comprehensive analysis of emotional vocabulary in the Headway series and propose a progressive teaching model that scaffolds emotional vocabulary instruction to support pre-service teachers’ socio-emotional and linguistic development.

### Theoretical framework: emotional intelligence theory

Goleman’s Emotional Intelligence (EI) Theory emphasizes the importance of emotional awareness, regulation, empathy, and social skills for personal and professional success, and in language education, EI is increasingly recognized as a crucial skill for effective teaching and learning. Goleman’s model provides a theoretical foundation for understanding how explicit instruction in emotion vocabulary can develop EI in pre-service EFL teachers (Guntersdorfer and Golubeva, 2018). Teaching emotional vocabulary equips future teachers with the tools to express, identify, and understand emotions, which are core components of EI. This instruction plays a crucial role in enhancing teachers’ self-awareness and their ability to cultivate both emotional and linguistic skills in learners. Research indicates that EI is essential for teacher effectiveness, helping educators navigate classroom challenges and foster positive learning environments (Sucaromana, 2012). Moreover, EI and social intelligence (SI) are recognized as core competencies for language teachers, with studies showing positive correlations between EI/SI levels and teaching experience (Mercer and Gkonou, 2017). Integrating EI into language teaching materials and practices promotes emotionally aware and socially competent teachers (Salovey and Sluyter, 1997), underscoring the need for explicit instruction in both pre-service and in-service programs to improve instructional performance and teacher retention (Corcoran and Tormey, 2012). Thus, incorporating EI and emotion vocabulary in EFL education not only enhances teacher effectiveness but also supports broader educational goals related to teacher well-being and student success.

### Conceptual framework

The conceptual framework for this study is designed to guide the investigation of emotion vocabulary in the *Headway* series and its potential to develop emotional intelligence (EI) in pre-service EFL teachers. It is structured around the relationship between emotion vocabulary, its categorization, and its role in fostering EI through teacher training programs. The independent variables include the emotion vocabulary in the *Headway* series, which will be measured by counting the emotion vocabulary words across all levels (Research Objective 1) and categorized by basic emotions (joy, anger, fear, sadness, surprise, and disgust), valence (positive, negative, neutral), and word class (verb, noun, adjective, adverb) (Research Objectives 2–4). The mediating variable is the systematic teaching model, which will be developed from the analysis of emotion vocabulary and will integrate emotion vocabulary instruction with emotional intelligence development (Research Objective 5). The dependent variable focuses on fostering emotional intelligence in pre-service EFL teachers, aiming to enhance their emotional awareness, regulation, and social skills, ultimately contributing to improved teaching effectiveness and classroom management. The conceptual flow begins with the identification and categorization of emotion words in EFL materials, leading to the development of an instructional model, which is then applied to foster emotional intelligence in pre-service teachers, resulting in better classroom management, communication, and student outcomes. This framework demonstrates the sequential relationship between emotion vocabulary analysis and its application to develop a systematic teaching model aimed at fostering EI in pre-service teachers.

### Literature review

Recent research has provided significant insights into how emotion vocabulary is lexicalized and its importance in language learning. Bąk (2022a) found that English predominantly lexicalizes emotions as adjectives, with negative emotions more frequently expressed as verbs and positive emotions as nouns, revealing the presence of the negative differentiation effect and the Pollyanna effect in both English and Polish (Bąk, 2022a, 2022b). In the context of EFL education, Sánchez and Pérez-García (2020) noted that newer EFL textbooks contain more emotion-laden words, reflecting a growing recognition of the role of emotional language in language acquisition. Lindquist (2017) further emphasized the crucial role of language in shaping emotion perception and experience. Additionally, socio-economic factors, such as emotioncy, have been found to influence vocabulary learning and retention, with highlighting the connection between socio-economic background and emotional experiences in vocabulary acquisition (Pishghadam and Shayesteh, 2016). Jiménez Catalán and Dewaele (2017) discovered that young Spanish EFL learners produced more words for non-emotion prompts than for emotion prompts, with positive prompts eliciting more words than negative ones, indicating a potential ease in engaging with positive emotional content. These findings underscore the complex relationship between emotion vocabulary, language learning, and emotional intelligence development, highlighting the need for a systematic approach to integrating emotional language in EFL education.

The categorization of emotions and emotional intelligence (EI) are closely related concepts in psychological research, both vital for emotional and social functioning. Emotions are often categorized into universal basic categories such as joy, anger, fear, sadness, and surprise, which are considered pancultural, while subordinate categories can be culture-specific (Russell, 1991; Shaver et al., 1987). These emotions can also be classified by valence, grouping them into positive, negative, or neutral categories. Positive emotions like joy are associated with beneficial effects on learning, while negative emotions such as anger and fear can impede emotional regulation. Salovey and Sluyter (1997) defined EI as the ability to perceive, express, understand, and regulate emotions in oneself and others, and is a set of skills that can be developed and linked to academic achievement (Götz et al., 2005). The development of EI is influenced by various factors, including motor, linguistic, and mental inference abilities (Hoemann et al., 2020), which are essential for effective emotion perception and categorization. Educational programs have been shown to foster EI, although they have limitations (Salovey and Sluyter, 1997). These programs often focus on improving learners’ ability to recognize and categorize emotions, which is critical for social competence and emotional regulation (Punia et al., 2015). Thus, understanding emotion categorization and developing EI are crucial for fostering emotional regulation, social competence, and overall well-being.

Emotion vocabulary spans multiple linguistic domains, including parts of speech, lexical semantics, and cultural categorization. Rijkhoff (2007) highlights typological variations in parts-of-speech systems across languages, which apply to emotion vocabulary, where words are classified into verbs, nouns, adjectives, and adverbs, each serving distinct semantic roles. Majid (2012) emphasizes that emotions interact with language at various structural levels, influencing how emotions are categorized and expressed. Soriano (2013) also stresses the importance of lexical semantics in understanding the function of emotion words in discourse. In the context of EFL materials, Sánchez and Pérez-García (2020) show that emotion vocabulary has been increasingly incorporated, with a focus on categorizing these words by part of speech for better language acquisition. Sh (2022) examines emotional verbs within English linguoculturology, while Wu and Zhang (2020) differentiate between emotion-label and emotion-laden words in affective neurolinguistics, highlighting their varied roles in conveying emotional content. Moreover, Russell (1991) explores cultural differences in emotion categorization, and Pennebaker et al. (2003) demonstrate that the use of function words, such as adjectives and adverbs, reveals deeper psychological and social aspects of individuals. These studies collectively underscore the significance of categorizing emotion vocabulary into parts of speech, influencing not only language learning but also emotional perception and cross-cultural communication.

Emotional intelligence (EI) plays a crucial role in teacher education, particularly in enhancing classroom management, pedagogical skills, and overall teaching effectiveness (Dick-Bursztyn, 2023). Research has demonstrated that EI significantly improves communication and creates effective learning environments, contributing to better language teaching and learning outcomes (Sucaromana, 2012). The correlation between EI and achievement in EFL classrooms is well-documented, with emotionally intelligent teachers showing greater success in managing both their own emotions and those of their students (Gharavi et al., 2013). This capacity is vital for improving teacher well-being, job performance, and student outcomes (Palomera et al., 2008). Teachers with high EI are better equipped to handle the complexities of the classroom, fostering positive environments and facilitating learning more effectively (Jiménez Catalán and Dewaele, 2017). Birwatkar (2014) states that the integration of EI into teacher education has the potential to transform educational systems, enhancing both student achievement and school effectiveness. Therefore, developing EI in pre-service teacher programs, especially in EFL contexts, is essential for improving emotional awareness and teaching efficacy.

Research on vocabulary content in EFL (English as a Foreign Language) textbooks has explored various aspects, including emotion vocabulary, high-frequency word coverage, and learning approaches. Sun and Dang (2020) examined vocabulary size requirements for comprehension, revealing significant variation across textbooks, with estimates suggesting that learners need between 3,000 and 6,000 word families for effective comprehension (Rahmat and Coxhead, 2021). While Catalán and Francisco (2008) highlighted the role of high-frequency vocabulary in language learning, Nakayama (2022) develop corpus-based analysis of Japanese EFL textbooks, and found that these textbooks covered a substantial number of high-frequency words as well as lacked sufficient coverage of lower-frequency bands. Additionally, Sánchez et al. (2022) showed that using imagination elicitation methods produced better results than context provision for learning emotional vocabulary. Xodabande et al. (2022) further underscored the need for contextualizing vocabulary to enhance learning outcomes. These findings collectively underscore the importance of balanced vocabulary coverage, including both high-frequency words and emotional vocabulary, to support comprehensive language learning in EFL textbooks.

While recent research has highlighted the significance of emotion vocabulary in language learning, particularly in fostering emotional intelligence (EI) and enhancing sociopragmatic competence, there remains a critical gap in the systematic inclusion and categorization of emotion vocabulary in widely used EFL materials such as the *Headway* series. Despite growing recognition of the importance of emotional language in EFL education, Dar (2018) indicate that many textbooks still underrepresent emotion vocabulary or fail to categorize it in ways that would facilitate language acquisition and emotional intelligence development. Furthermore, although some research has explored the emotional and psychological effects of emotion-laden texts (Driver, 2022), there has been limited focus on how this vocabulary can be systematically integrated into pre-service EFL teacher training programs to foster EI. This gap necessitates an in-depth investigation into the presence and categorization of emotion vocabulary in the *Headway* series, with the aim of using these findings to develop effective teaching models for EI in EFL contexts.

## Methods

### Research design

This study adopted a mixed-methods approach, integrating both qualitative and quantitative research methods to provide a comprehensive understanding of the role of emotion vocabulary in fostering emotional intelligence (EI) among pre-service EFL teachers. This combination allows for a robust analysis, leveraging the strengths of both approaches to offer a richer dataset and more nuanced insights (Creswell and Clark, 2017). The quantitative aspect focused on systematically measuring and categorizing emotion vocabulary across the Headway series using established lexicons such as the EMCAT-ENG (Bąk, 2022a) and Warriner’s affective word list of valence, arousal and dominance (Warriner et al., 2013). Emotion-related words were counted and classified by basic emotions (joy, anger, fear, sadness, surprise, and disgust), valence (positive, negative, neutral), and word class (verb, noun, adjective, adverb). This quantitative analysis provided numerical data to identify trends and gaps in the presence of emotion vocabulary across the textbooks. The qualitative component involved a thematic analysis that aligned emotion vocabulary with key emotional intelligence components, such as self-awareness, emotional regulation, and empathy, which are essential for pre-service teachers (Goleman, 2020). This mixed-methods design offers objective measures of vocabulary inclusion through quantitative data while providing deeper insights into how this vocabulary can be pedagogically aligned with emotional intelligence to foster more effective teaching outcomes (Tashakkori, 1998).

### Data source

In this study, data were systematically collected from the Headway Vocabulary Wordlist, which is meticulously aligned with the units and themes of the corresponding textbooks. These vocabulary words were carefully curated to match the proficiency levels of learners, spanning six levels: beginner, elementary, pre-intermediate, intermediate, upper-intermediate, and advanced. The wordlists covered a wide range of topics such as travel, work, and relationships, and were organized by unit and theme to ensure comprehensive coverage across all textbook levels. Each wordlist is associated with the widely recognized Headway English as a Foreign Language (EFL) series, which is not only esteemed for its effectiveness in enhancing language acquisition and boosting learners’ confidence but also widely used to train pre-service English teachers across the world, including Chile. The textbook series has been the subject of numerous studies, including those exploring Sustainable Development Goals (SDG) content (Lasekan et al., 2023). The wordlists included various word types—nouns, verbs, adjectives, and adverbs—accompanied by definitions and, in some cases, phonetic transcriptions to facilitate accurate pronunciation. Additionally, contextual examples of word usage in sentences were provided to aid learners in understanding both meaning and application. Common collocations and useful phrases were also included, enabling learners to use vocabulary naturally and fluently in both spoken and written communication. This comprehensive wordlist serves as a valuable resource for reinforcing vocabulary acquisition and supporting both retention and practical application, playing a vital role in the overall learning process within the Headway series.

### Data analysis

#### Quantitative analysis

The quantitative aspect of the methodology focused on systematically measuring the frequency and categorization of emotion vocabulary within the Headway series. By employing established lexicons such as EMCAT-ENG (Bąk, 2022a) and Warriner’s affective word list (Warriner et al., 2013), emotion words were classified by basic emotions (e.g., joy, sadness, anger), valence (positive, negative, neutral), and part-of-speech (verb, noun, adjective, adverb). Statistical analyses, conducted using tools like Atlas.ti, allowed for descriptive insights into the distribution and trends of emotion vocabulary across proficiency levels. This quantitative analysis offers objective, replicable data, identifying gaps and trends in the inclusion of emotion vocabulary within the textbooks.

#### Qualitative analysis

The qualitative component complemented the quantitative findings by conducting a thematic analysis that linked identified emotion vocabulary to key EI components such as self-awareness, emotional regulation, and empathy (Goleman, 2020). This analysis provided deeper insights into the pedagogical potential of emotion vocabulary, highlighting its relevance to fostering EI among pre-service teachers. The qualitative analysis moved beyond numerical trends to explore the contextual and instructional significance of these words in real-world teaching scenarios.

Thus, this mixed-methods approach is not only sequential but also complementary. This approach allows researchers to explore different dimensions of a research question, leading to more rigorous and comprehensive insights (Cisneros-Barahona et al., 2024). The quantitative data provided a foundational understanding of the prevalence and distribution of emotion vocabulary, while the qualitative analysis interprets these findings in the context of emotional intelligence and pedagogy. By combining the strengths of quantitative precision and qualitative depth, the mixed-methods design provides a holistic framework for exploring how emotion vocabulary can be systematically incorporated into teacher training to enhance emotional intelligence and pedagogical effectiveness.

### Reliability and validity

To ensure reliability, inter-coder agreement was employed during the content and thematic analyses. Multiple researchers independently categorized emotion vocabulary by basic emotions (e.g., joy, sadness), valence (positive, neutral, negative), and word class (verb, noun, adjective, adverb). Discrepancies were resolved through discussions, ensuring consistency and reducing bias. This approach provided a robust and replicable dataset for analyzing the Headway series.

For validity, the systematic teaching model was reviewed through expert evaluations and focus group discussions. Expert reviews ensured alignment with Goleman’s Emotional Intelligence framework and practical relevance in teacher training, while focus groups with pre-service teachers assessed the model’s applicability and effectiveness.

## Results

**Figure 1:**
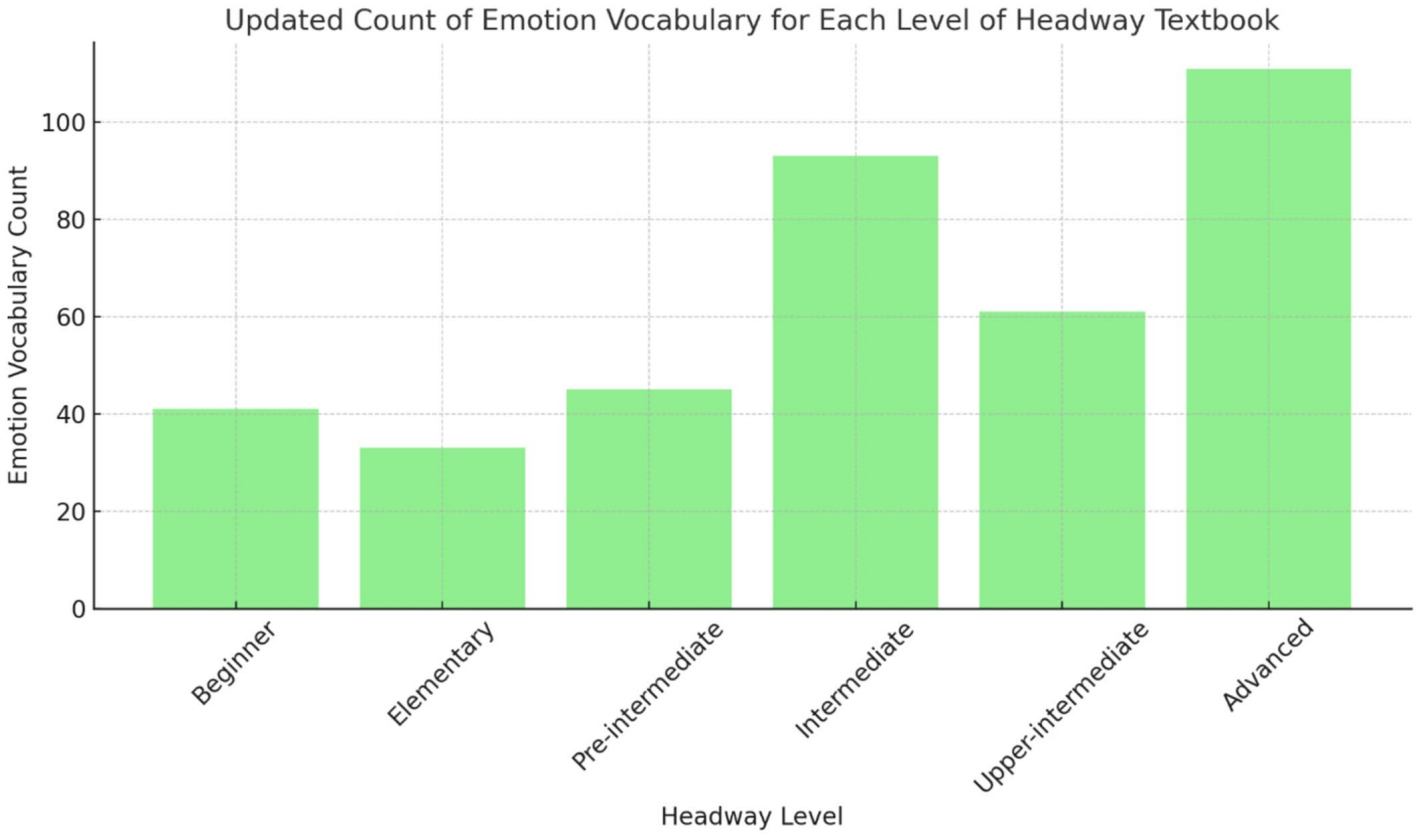
Emotion vocabulary count across proficiency levels in the headway series.

The findings for Research Objective 1, which sought to quantify the emotion vocabulary across all levels of the Headway series, reveal a progressive increase in emotional vocabulary as proficiency levels advance. At the beginner and elementary levels (Figure 1), the count is relatively low, with approximately 40 words at the beginner stage and slightly fewer at the elementary level. However, the pre-intermediate and intermediate levels show a marked increase, particularly at the intermediate level, where the count rises to nearly 90 words. The most significant growth occurs at the advanced level, where the number of emotion vocabulary words exceeds 100. This progression reflects a deliberate pedagogical strategy, gradually introducing more complex emotional language as learners become more proficient. The lower counts at the early stages indicate a potential gap in early exposure to emotion vocabulary, suggesting that greater emphasis on emotional expressions at these levels could enhance emotional intelligence development in learners.

**Figure 2:**
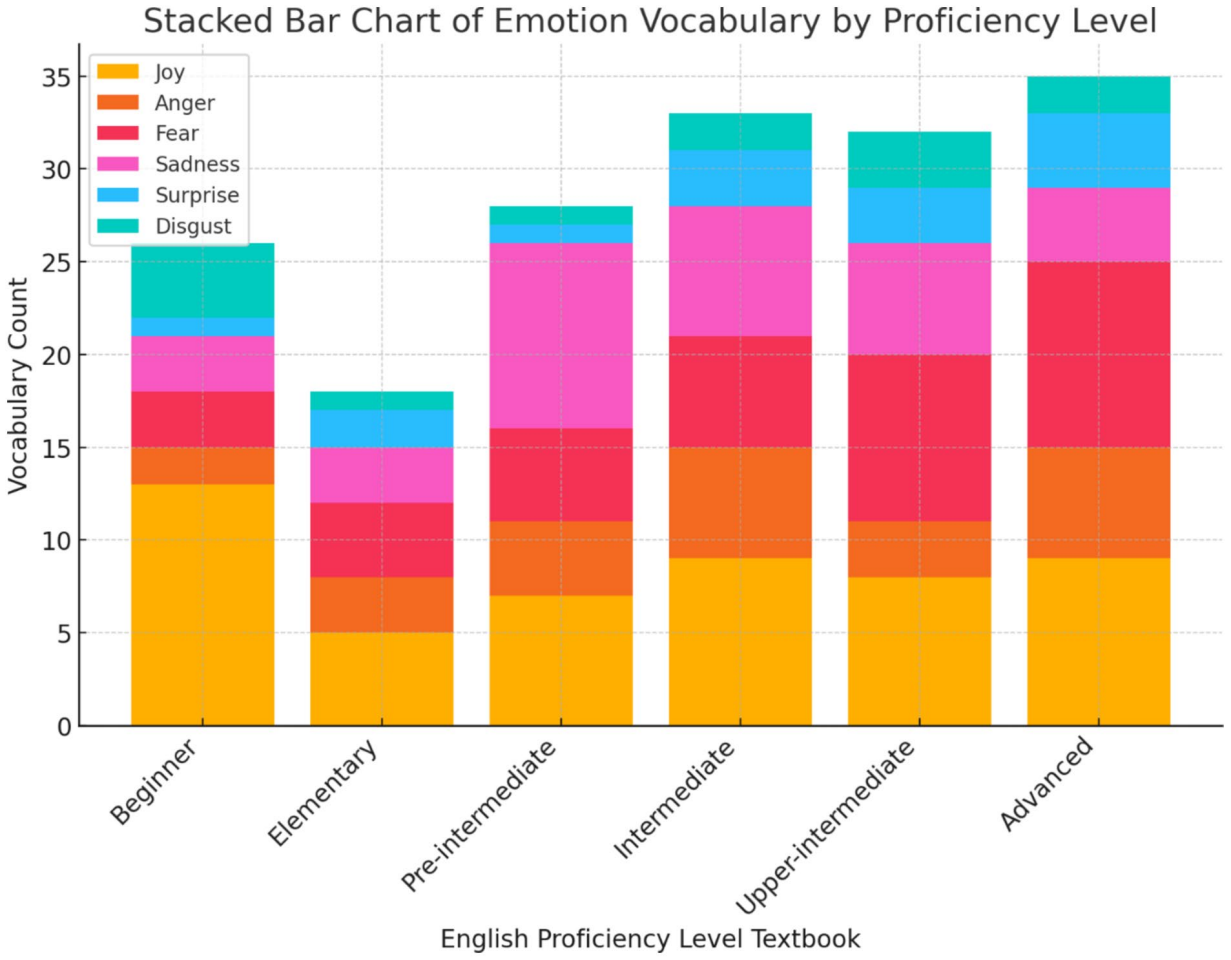
Categorization of basic emotions in the headway series across proficiency levels.

The results for Research Objective 2, which focused on categorizing and quantifying emotion vocabulary into basic emotions (joy, anger, fear, sadness, surprise, and disgust) across all levels of the *Headway* series (Figure 2), reveal distinct trends. Joy is the most frequent emotion across all levels, particularly prominent at the beginner level, suggesting a focus on positive emotions early in language learning to build confidence. Anger and fear are moderately represented at all levels, with a noticeable increase at intermediate and advanced stages, reflecting the gradual introduction of more complex and negative emotions as proficiency grows. Sadness shows a steady presence across levels, with a significant rise at the higher stages, indicating its role in advanced sociopragmatic competence. Surprise and disgust are less represented overall but increase slightly at the upper-intermediate and advanced levels, highlighting their complexity and later introduction in emotional vocabulary development. These findings suggest a structured progression of emotional vocabulary, with simpler, positive emotions introduced at lower levels and more complex, negative emotions reserved for advanced learners.

**Figure 3:**
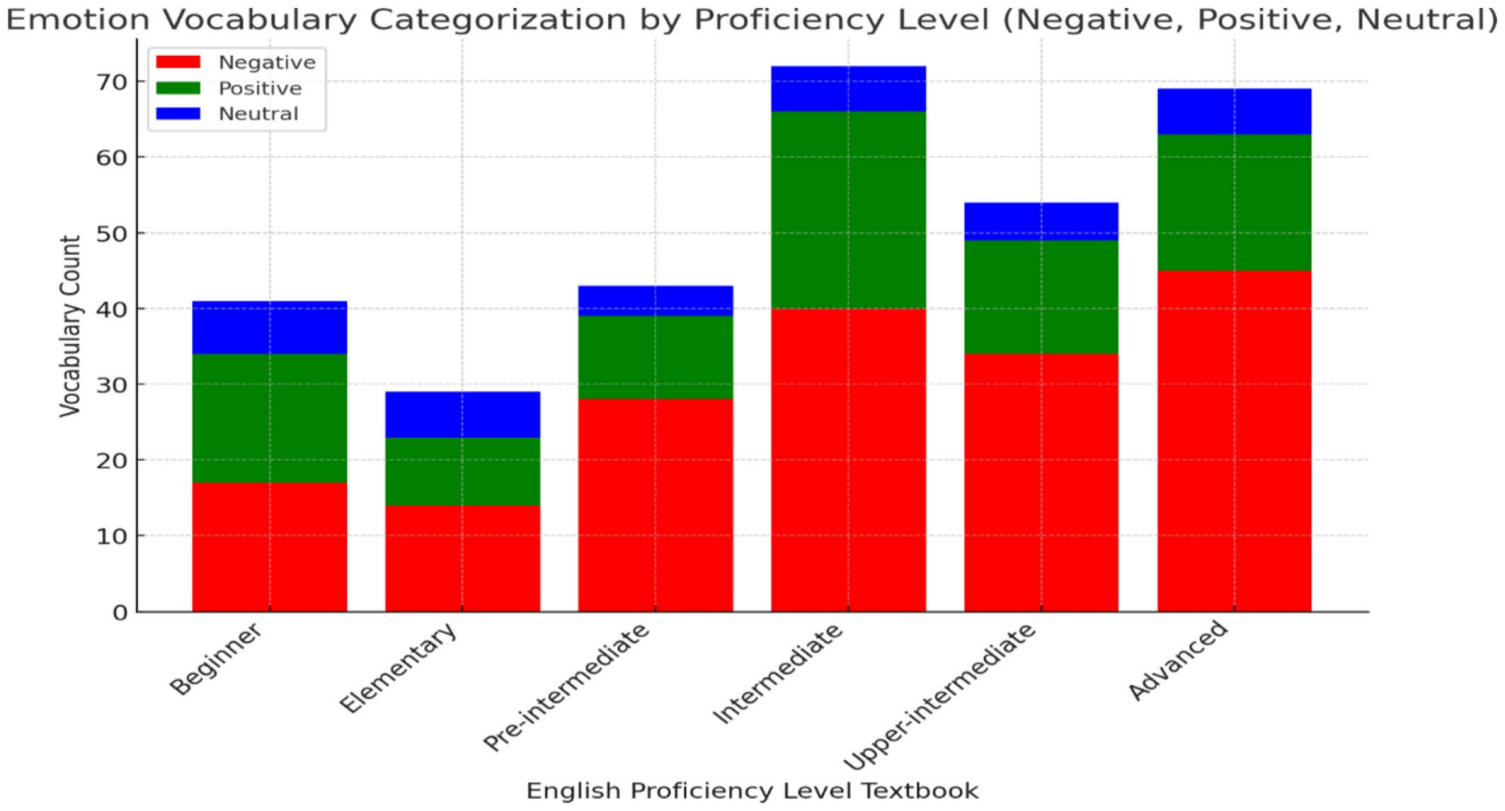
Emotion vocabulary categorization by valence across proficiency levels in the headway series.

The findings for Research Objective 3, which aimed to classify and quantify emotion vocabulary by valence (positive, negative, neutral) across all levels of the *Headway* series (Figure 3), reveal that negative emotion vocabulary is dominant across all proficiency levels, from beginner to advanced. This suggests a strong focus on teaching learners to express and understand negative emotions, which are essential for real-life communication and emotional literacy. Positive emotion vocabulary is consistently present but less frequent, with a slight increase at intermediate and advanced stages, indicating an emphasis on handling complex emotional communication involving both positive and negative contexts. Neutral emotion vocabulary, while the least represented, progressively increases at higher proficiency levels, particularly at intermediate and advanced stages, suggesting its role in facilitating nuanced emotional expressions as learners’ language skills develop. These findings reflect a pedagogical strategy that prepares learners to navigate both positive and negative emotional interactions while gradually introducing more subtle, neutral emotions in advanced communication scenarios.

**Figure 4:**
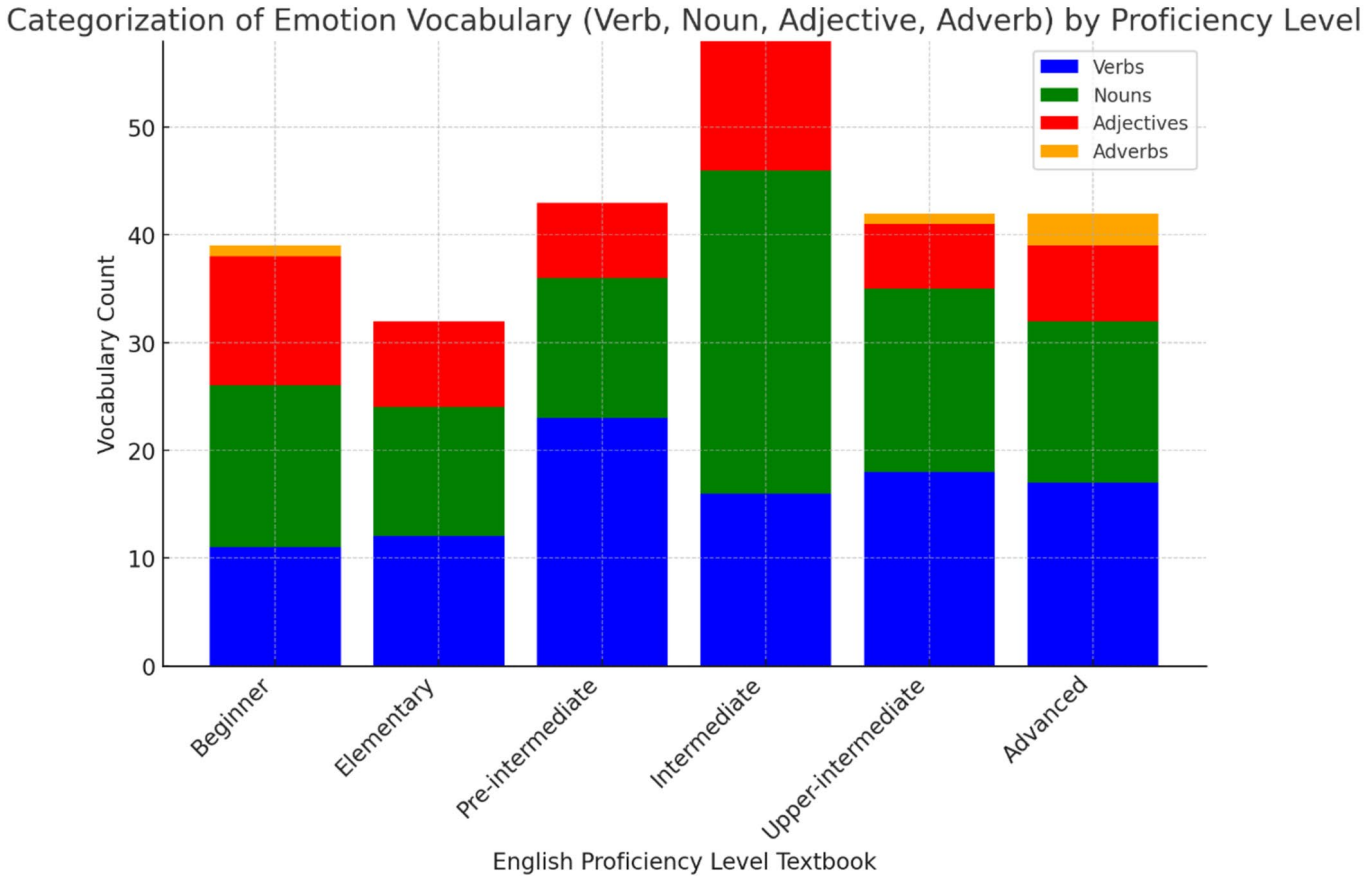
Categorization of emotion vocabulary by word class across proficiency levels in the headway series.

Research Objective 4, which sought to categorize and quantify emotion vocabulary by word class (verb, noun, adjective, adverb) across all levels of the *Headway* series (Figure 4), indicate that verbs and nouns dominate emotional vocabulary across all proficiency levels. Verbs, consistently represented, highlight the importance of action-oriented emotional expressions, while nouns, particularly prominent at the intermediate level, allow learners to discuss emotions more abstractly. Adjectives play a significant role, especially at higher levels, helping learners describe emotions in nuanced ways. Adverbs are the least represented word class, with their frequency increasing at the upper-intermediate and advanced stages, suggesting they are introduced later to refine emotional expression. This progression demonstrates a structured approach, starting with fundamental emotional vocabulary and gradually incorporating more complex descriptive language as learners advance in proficiency.

### Systematic vocabulary teaching model to foster emotional intelligence in pre-service EFL teachers

Emotional intelligence (EI) is increasingly recognized as a crucial skill for teachers, particularly in language education, as it enhances classroom management, communication, and learning outcomes (Marashi and Zaferanchi, 2010). Pre-service EFL teachers, in particular, benefit from EI development as it equips them to manage their emotions and fosters empathy and motivation in their students (Turner and Stough, 2020). One effective way to foster EI in pre-service teachers is through systematic vocabulary instruction that aligns with the key components of EI: self-awareness, self-regulation, social skills, motivation, and empathy (Goleman, 2020). This paper proposes a teaching model based on findings from the Headway series, aimed at progressively introducing emotional vocabulary to support the development of EI in pre-service EFL teachers.

### Proposed model overview

**Figure 5:**
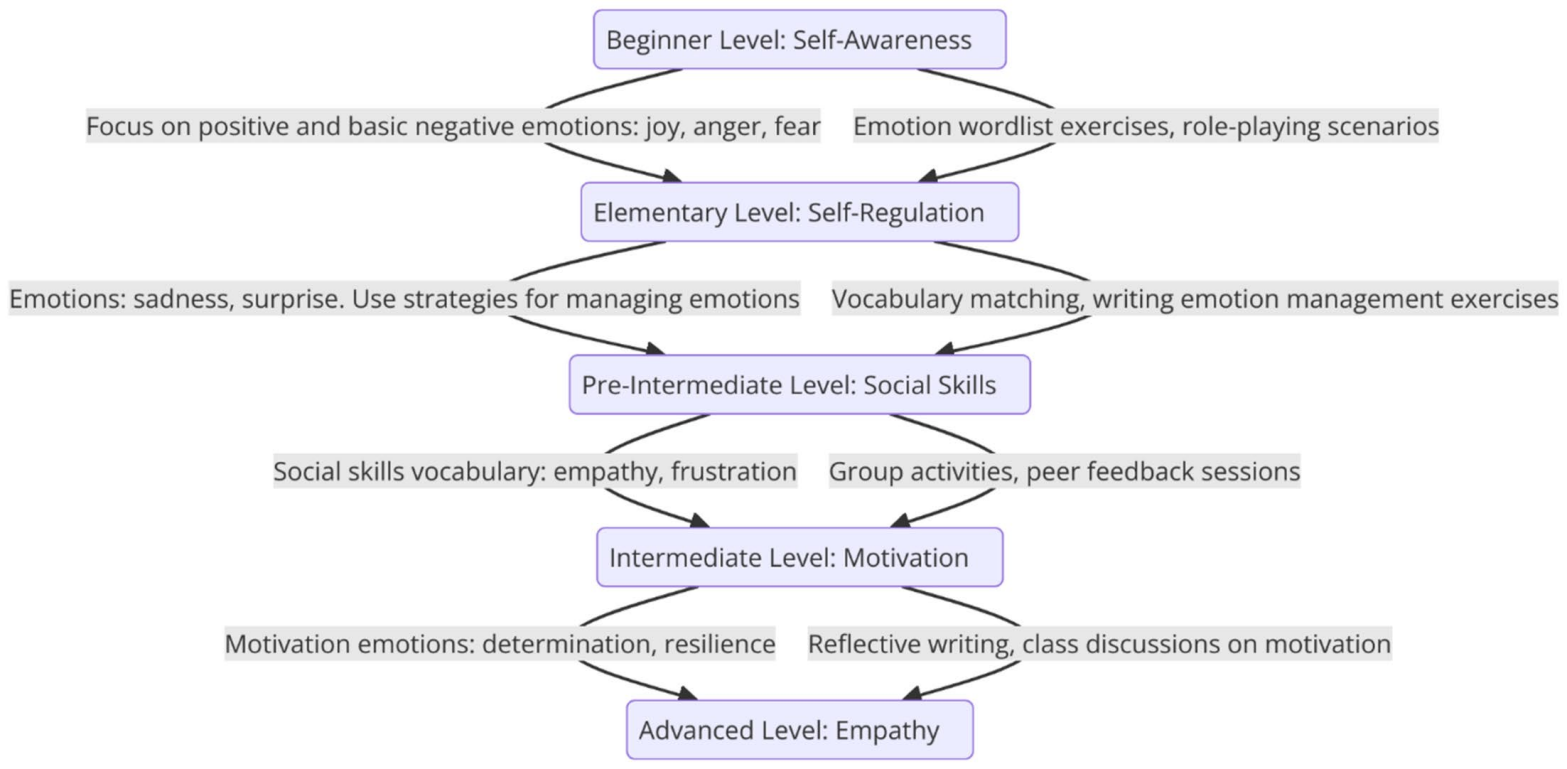
Scaffolded emotional vocabulary teaching model for enhancing emotional intelligence in pre-service EFL teachers.

The development of the Systematic Vocabulary Teaching model leverages Design-Based Research (DBR) methodology, which enables the iterative design and refinement of educational interventions through analysis, implementation, and evaluation (Tweeten and Hung, 2023). As shown in Figure 5, the model is structured around a scaffolded approach to vocabulary instruction (Booth, 2014), progressively introducing and expanding emotional vocabulary across five proficiency levels: beginner, elementary, pre-intermediate, intermediate, and advanced. Using DBR, the sequence of these proficiency levels was informed by empirical findings from the Headway series and aligned with theoretical frameworks such as Goleman’s Emotional Intelligence (EI) Theory. Emotional vocabulary focusses and corresponding EI components were systematically mapped to each level, ensuring alignment with learners’ linguistic and socio-emotional development. Each level addresses specific EI components—such as self-awareness, self-regulation, and empathy—helping pre-service teachers build emotional literacy and develop the emotional intelligence needed for effective teaching. Activities, such as role-playing, vocabulary matching, and reflective journaling, were co-designed with educators, piloted, and refined based on real-world feedback to optimize engagement and outcomes. Each level’s justification was grounded in both theoretical insights and classroom observations, ensuring that the model remained practical, effective, and contextually relevant.

#### Beginner level (focus: self-awareness)

- Vocabulary Focus: Positive and basic negative emotions (e.g., joy, anger, fear).
- EI Component: Self-awareness—At the beginner level, teachers need to recognize and name their own emotions and those of their learners.

Activities:

o Emotion wordlist exercises focused on identifying basic emotions (e.g., “What makes you feel joy?”).
o Role-playing scenarios where teachers identify the emotions in conversations.

- Justification: At this stage, developing self-awareness is critical for personal emotional intelligence and future emotional regulation. Recognizing emotions helps teachers understand their emotional states, a foundational skill in EI (Goleman, 2020).

#### Elementary level (focus: self-regulation)

- Vocabulary Focus: Adjectives and verbs for describing emotions (e.g., sadness, surprise).
- EI Component: Self-regulation—Teachers learn to regulate emotions in different classroom scenarios.

Activities:

o Vocabulary matching tasks linking emotions with appropriate classroom responses (e.g., “If you feel anger, how would you react?”).
o Writing exercises that describe emotions and propose strategies for managing them (e.g., “When I feel frustrated, I…”).

- Justification: Self-regulation is essential for managing emotions, especially in stressful situations. Expanding emotional vocabulary helps teachers describe and manage their emotional responses (Hall and Goetz, 2013).

#### Pre-intermediate level (focus: social skills)

- Vocabulary Focus: Broader range of adjectives, including neutral emotional vocabulary (e.g., empathy, frustration).
- EI Component: Social skills—Teachers develop the ability to communicate emotions effectively in social settings.

Activities:

o Group activities where learners use emotion vocabulary to describe social interactions and resolve conflicts (e.g., “How would you express empathy in this situation?”).
o Peer feedback sessions evaluating emotional communication during role-playing tasks.

- Justification: Social skills are crucial for effective classroom management and communication. Expanding emotional vocabulary helps teachers navigate social interactions and build rapport with students (Alzina and Guiu, 2018).

#### Intermediate level (focus: motivation)

- Vocabulary Focus: Expanded emotion vocabulary, including motivational and goal-oriented emotions (e.g., determination, resilience).
- EI Component: Motivation—Understanding and expressing motivation helps pre-service teachers stay committed to their goals.

Activities:

o Reflective journal writing describing how emotions like frustration and determination impact teaching practice.
o Class discussions exploring motivation using emotion vocabulary to discuss persistence in challenging situations.

- Justification: Motivation-related emotional vocabulary helps teachers inspire themselves and their students, fostering perseverance and goal-setting (Hall and Goetz, 2013).

#### Advanced level (focus: empathy)

- Vocabulary Focus: Complex emotional expressions related to empathy and understanding others (e.g., regret, compassion).
- EI Component: Empathy—Teachers develop the ability to recognize and respond empathetically to learners’ emotions.

Activities:

- o Advanced role-playing scenarios where learners use empathy-related vocabulary to respond to emotional classroom situations.
- o Reflective writing analyzing classroom cases involving student emotions and proposing empathetic responses.

- Justification: Empathy is key for creating supportive learning environments. Mastering emotion vocabulary that conveys empathy enables teachers to foster emotional connections with their students, enhancing learning outcomes (Mercer, 2016).

The systematic teaching model for fostering emotional intelligence (EI) in pre-service EFL teachers is directly shaped by the findings from the *Headway* series, which reveal a progressive increase in emotional vocabulary complexity across proficiency levels. Simpler emotions like joy and fear are introduced at the beginner level to build self-awareness, while more complex emotions such as empathy and frustration are integrated at advanced levels to foster social skills and empathy. The focus on positive emotions in the early stages supports the development of self-awareness, while the inclusion of more nuanced negative emotions at advanced levels aids in fostering empathy and managing complex classroom interactions. Additionally, the prominence of negative emotions across all levels emphasizes the need for self-regulation and motivation in the elementary and intermediate stages, helping teachers learn to identify and manage emotions effectively. As learners progress, the introduction of neutral emotional vocabulary enhances their ability to express emotions with subtlety, which is crucial for empathy. The categorization of emotion vocabulary by word class—where verbs and nouns dominate at lower levels, and adjectives and adverbs increase at higher levels—further informs vocabulary activities that develop social skills and empathy. These insights ensure that the model aligns with the progressive development of emotional intelligence, equipping pre-service teachers with the emotional and linguistic tools necessary for effective classroom management and improved student outcomes.

## Discussion

Counting the number of emotion vocabulary words across all levels of the *Headway* series showed a clear progression in emotional vocabulary as proficiency levels increase. This aligns with prior research emphasizing the role of emotion vocabulary in both language acquisition and the development of emotional intelligence (Sánchez and Pérez-García, 2020). While the introduction highlighted a general scarcity of emotion vocabulary in EFL materials (Dewaele, 2015), the results indicate that the *Headway* series employs a deliberate strategy to gradually introduce more complex emotional language at higher proficiency levels, which supports emotional intelligence growth. This gradual increase reflects emotional intelligence theory, where exposure to simpler emotions at earlier stages helps build self-awareness and social competence over time (Goleman, 2020). However, the relatively low inclusion of emotion vocabulary at the beginner and elementary levels raises concerns about early exposure, mirroring criticisms in the literature that call for greater emphasis on emotional language at lower proficiency levels to support early emotional intelligence development (Alzina and Guiu, 2018). Thus, while the *Headway* series demonstrates improvements, the findings suggest a need for stronger focus on emotional vocabulary inclusion at the initial stages to fully realize the potential of emotional intelligence teaching in EFL contexts.

Addressing findings related to Research Objective 2, which focused on categorizing and quantifying emotion vocabulary within the Headway series according to the six basic emotions: joy, anger, fear, sadness, surprise, and disgust. The analysis reveals notable patterns in the distribution of these emotions, providing insights into how emotional language is represented across the series. Joy emerged as the most frequent emotion, particularly at the beginner level, which aligns with the introduction’s emphasis on positive emotions as foundational for building learner confidence (Sánchez and Pérez-García, 2020). The lower representation of complex negative emotions, such as disgust, at early proficiency levels supports the literature’s suggestion that advanced learners are better equipped to handle nuanced emotional language (Bąk, 2022b). The gradual introduction of more complex negative emotions at intermediate and advanced stages reflects the progressive development of emotional vocabulary, which parallels learners’ linguistic growth and sociopragmatic competence (Dewaele, 2015). While emotions such as joy and sadness are relatively balanced across levels, the late inclusion of emotions like surprise and disgust indicates a deliberate effort to introduce more challenging emotional vocabulary only after learners have built a stronger linguistic foundation. This finding supports the idea that negative emotions play a significant role in vocabulary retention and emotional intelligence development at higher levels (Driver, 2022). However, the limited presence of negative emotions at the beginner and elementary stages raises concerns about the early development of emotional literacy, reinforcing the literature’s call for a more comprehensive integration of emotional vocabulary from the start to enhance learners’ emotional awareness (Alzina and Guiu, 2018).

One of the objectives of this study was to classify and quantify emotion vocabulary by valence (positive, negative, neutral) across all levels of the *Headway* series, revealed a dominance of negative emotion vocabulary throughout the proficiency levels. This aligns with Driver’s (2022) claim that exposure to negative and neutral emotion-laden texts enhance vocabulary retention and supports better learning outcomes. However, this finding contrasts with the introduction’s emphasis on the importance of positive emotions, particularly in early language acquisition (Sánchez and Pérez-García, 2020). Although positive emotions are present across all levels, their frequency remains lower compared to negative emotions, especially at intermediate and advanced stages, which suggests a pedagogical focus on preparing learners for more complex emotional communication. The gradual introduction of neutral emotion vocabulary at higher proficiency levels is consistent with the literature, which indicates that neutral emotional expressions gain importance as learners’ fluency increases (Bąk, 2022b). While the prominence of negative emotion vocabulary may support advanced language acquisition, the balance between positive and neutral emotions could be improved at lower levels to foster early emotional intelligence development (Dewaele, 2015), which is crucial for pre-service teachers to manage emotions in educational contexts.

Categorizing and quantifying emotion vocabulary by word class (verb, noun, adjective, adverb) across all levels of the *Headway* series revealed that verbs and nouns dominate the emotional vocabulary throughout all proficiency levels. This aligns with the literature’s emphasis on the role of verbs in facilitating action-oriented emotional expressions and nouns in enabling more abstract emotional discussions (Majid, 2012). However, adjectives, which are critical for precise emotional descriptions, only become prominent at higher levels, potentially limiting learners’ ability to describe emotions with nuance in earlier stages (Rijkhoff, 2007). The lower representation of adverbs, especially at lower levels, suggests a gap in providing learners with the tools to express emotions with subtlety, a skill important for advanced communication (Dar, 2018). While the structured progression of emotional vocabulary development aligns with theoretical frameworks from the literature, the findings highlight the need for a more balanced inclusion of word classes, particularly at earlier levels, to enhance learners’ ability to express and regulate emotions from the start of their language learning journey.

This investigation focused on developing a systematic teaching model to foster emotional intelligence (EI) in pre-service EFL teachers, align with the theoretical foundations discussed in the literature but also reveal areas for improvement. The model’s progressive integration of more complex emotional vocabulary as proficiency increases reflects Goleman’s (2020) theory of emotional intelligence, where emotional awareness and regulation are built gradually. This approach is consistent with Sánchez and Pérez-García’s (2020) emphasis on the importance of emotion-laden vocabulary for enhancing both language acquisition and EI development. However, despite the model’s effectiveness in scaffolding emotional vocabulary instruction across proficiency levels, the underrepresentation of emotional vocabulary at the beginner and elementary stages, as noted in earlier findings, may limit the early development of self-awareness and emotional regulation in pre-service teachers. This gap suggests a need for more balanced inclusion of emotional vocabulary across all levels to ensure early exposure to emotional concepts, which is crucial for the development of EI from the outset (Dewaele, 2015). While the model holds significant potential, addressing these gaps would enhance its effectiveness in fostering emotional intelligence in future educators.

Enhanced emotional intelligence (EI) in pre-service EFL teachers, as fostered by the proposed systematic vocabulary teaching model, can significantly improve classroom management, communication, and student outcomes. Research indicates that emotionally intelligent teachers are better equipped to manage the emotional dynamics of the classroom, leading to more effective classroom management and a more supportive learning environment (Goleman, 2020). Teachers with strong EI skills, such as self-awareness and emotional regulation, are able to communicate more empathetically and clearly with their students, which fosters trust and rapport, key elements for successful learning experiences (Coombe et al., 2020). Additionally, the ability to recognize and respond to students’ emotional states allows teachers to address emotional barriers to learning, ultimately improving student engagement and performance (Sun et al., 2024). The model’s emphasis on progressively building emotional vocabulary helps teachers develop these essential skills, which are critical not only for their professional well-being but also for promoting positive student outcomes, such as increased motivation and language proficiency. Consequently, the integration of emotional intelligence through language instruction supports both teacher effectiveness and student success in EFL contexts.

The theoretical and conceptual frameworks in this study play a pivotal role in guiding the research and ensuring its alignment with existing literature on emotional intelligence (EI) and language acquisition. Goleman’s Emotional Intelligence Theory serves as the theoretical foundation, emphasizing the importance of emotional awareness, regulation, empathy, and social skills. This framework justifies the need to incorporate emotional vocabulary into EFL teaching, aligning with prior research that underscores the role of EI in enhancing classroom management, communication, and student outcomes (Mercer, 2016). The conceptual framework further connects the study’s objectives by outlining how emotion vocabulary is categorized and measured across the *Headway* series and how these findings inform the development of a systematic teaching model. This clear relationship between emotion vocabulary and EI development reflects the literature’s call for a more structured integration of emotional concepts into language teaching (Sánchez and Pérez-García, 2020; Dewaele, 2015). Ultimately, these frameworks ensure that the study is methodologically sound and contribute to advancing both EI and language pedagogy, providing a solid foundation for enhancing pre-service teachers’ professional efficacy and emotional competence.

The primary advantage of the proposed Systematic Vocabulary Teaching model lies in its direct focus on developing emotional literacy and emotional intelligence (EI) skills among pre-service teachers, positioning them at the core of the intervention while also acknowledging the indirect benefits for their students. By emphasizing their self-awareness, self-regulation, and empathy, the model directly supports their ability to navigate complex classroom dynamics, manage stress, and foster positive relationships with students (Jiang et al., 2022). This targeted focus ensures alignment with the model’s primary objective of equipping educators with the skills necessary for effective classroom management and communication. However, the indirect impact on students is also significant, as emotionally literate teachers are better positioned to create supportive learning environments that enhance student engagement and well-being (Gabryś-Barker, 2018). While the model primarily evaluates teacher outcomes, tools like classroom climate surveys or the Classroom Assessment Scoring System could further capture these secondary effects, emphasizing both teacher and student development (Adams et al., 2021).

The systematic and progressive teaching model proposed in this study holds significant potential for adaptation to other EFL teaching materials beyond the Headway series, offering broader applicability across diverse educational contexts. The model’s core framework, which scaffolds emotional vocabulary instruction from simple to complex and aligns it with socio-emotional competencies, can be tailored to fit the structure and thematic focus of various EFL resources. For instance, textbooks with a business English focus could emphasize vocabulary related to professional emotional contexts, such as negotiation or conflict resolution (Cui et al., 2021), while materials for academic English could prioritize emotions tied to critical thinking and collaboration (Mestre-Mestre, 2020). Additionally, the model’s flexibility allows for cultural and demographic adaptations, ensuring alignment with specific learner needs and socio-cultural norms. To facilitate this broader application, practical guidelines can be developed, such as identifying and categorizing emotional vocabulary from alternative materials and mapping it to appropriate emotional intelligence competencies. By incorporating such adaptability, the proposed model ensures its relevance for educators working with a wide range of teaching resources, fostering emotional intelligence development in diverse learning environments.

Evaluating the impact of the proposed scaffolded model on the development of teachers’ emotional literacy and emotional intelligence (EI) is essential for understanding the effective implementation of the model. By employing pre-and post-assessments, reflective journals, and role-play evaluations, the model provides a comprehensive framework for tracking progress in self-awareness, self-regulation, and empathy (Alemdar and Anilan, 2020). Reflective journals reveal changes in teachers’ capacity to identify and regulate emotions effectively, aligning with the Emotional Literacy Skills Scale’s validated components (Haghani et al., 2014). Role-play scenarios and simulation feedback demonstrate real-time evidence of applying emotional vocabulary and managing classroom dynamics, reinforcing the measurable benefits of emotional competence training (Fincias et al., 2018). Moreover, a focus on teacher and learner interactions supports the promotion of emotional awareness as essential for fostering collaboration and achieving educational goals (Herguner, 2017). By systematically evaluating each competency with these diverse tools, the model not only aligns with theoretical frameworks but also ensures its practical relevance in real-world teaching contexts (Alemdar and Anilan, 2020).

The findings of this study underscore the importance of considering cultural factors in the pedagogical application of emotional vocabulary teaching. Emotional expressions are deeply rooted in cultural norms and values, influencing how emotions are categorized, prioritized, and taught in educational materials. For example, cultures with collectivist orientations, such as Chile, may emphasize emotional vocabulary related to social harmony and empathy (Kikutani et al., 2024), while individualistic cultures may prioritize vocabulary reflecting personal achievement and self-expression (Imada, 2012). This cultural variability suggests that emotional vocabulary in EFL textbooks like the Headway series may not fully address the needs of diverse learner populations. The proposed teaching model bridges this gap by incorporating cultural awareness into vocabulary instruction, encouraging educators to adapt emotional vocabulary teaching to reflect the sociocultural contexts of their learners. Activities such as reflective journaling and role-playing can be tailored to highlight culturally relevant emotions, fostering both linguistic proficiency and intercultural competence (Russell, 1991). By integrating cultural sensitivity, the model ensures that emotional vocabulary instruction aligns with the diverse emotional frameworks of learners, enhancing its relevance and effectiveness in fostering emotional intelligence.

Based on the findings and discussion in this study, several recommendations emerge for teachers, higher education institutions, and policymakers. For teachers, it is crucial to integrate emotional vocabulary instruction within the EFL curriculum at all proficiency levels to foster emotional intelligence (EI) in students. Educators should employ strategies that enhance self-awareness, emotional regulation, and empathy, facilitating not only language acquisition but also emotional literacy (Coombe et al., 2020). Higher education institutions should incorporate EI training into pre-service teacher education programs, ensuring that future educators are well-equipped with both linguistic and emotional skills necessary for managing classroom dynamics and improving student outcomes (Goleman, 2020). This training can include reflective practices, role-playing, and emotional vocabulary instruction to help students develop critical emotional competencies. Policymakers should consider revising teacher education curricula to mandate the inclusion of emotional intelligence development, recognizing its impact on teacher well-being, student engagement, and overall educational success (Mercer and Gkonou, 2017). Policies promoting EI-focused professional development for in-service teachers should also be encouraged to continuously improve teaching effectiveness and emotional awareness in the classroom. These recommendations ensure a holistic approach to language education, integrating emotional and linguistic development for both teachers and students.

## Conclusion

This study aimed to investigate the role of emotional vocabulary in fostering emotional intelligence (EI) in pre-service EFL teachers, with a specific focus on the Headway series textbooks. The general objective was to count, categorize, and classify emotion vocabulary across different proficiency levels while using the findings to develop a systematic teaching model for enhancing EI in pre-service teachers. The findings revealed a progressive increase in emotion vocabulary across proficiency levels, with more complex emotions and word classes introduced at higher stages. While positive emotions were more prominent at the beginner level, negative and neutral emotions increased in frequency at advanced stages, supporting the development of self-regulation and empathy.

The findings also have practical implications for teacher training programs. It is recommended that emotional vocabulary and EI development be incorporated into pre-service teacher education, preparing future educators to navigate the emotional complexities of the classroom. Teacher education programs should include specific training modules on emotional intelligence, such as reflective practices, role-playing, and emotional vocabulary instruction. Such training would help pre-service teachers develop key EI skills, including self-awareness, empathy, and emotional regulation, which are essential for maintaining positive classroom environments and improving student engagement.

Despite these valuable insights, several limitations were identified. First, the analysis was restricted to the Headway series, which may not fully represent the diversity of EFL teaching materials used worldwide. Additionally, the study focused primarily on the quantitative aspects of emotional vocabulary without fully exploring the contextual nuances of how these emotions are taught or interpreted by learners. Future research should expand the scope to include other textbook series and incorporate more qualitative analyses that explore how pre-service teachers engage with emotional vocabulary in real-life teaching scenarios.

Future work could also explore the long-term impact of systematic emotional vocabulary instruction on both teacher efficacy and student outcomes, particularly in diverse cultural and linguistic contexts. Moreover, further studies could develop and test emotional intelligence-focused curricula that integrate emotional vocabulary with reflective practices, role-playing, and classroom discussions to assess their effectiveness in fostering EI in pre-service teachers across different teaching environments. Overall, the study underscores the importance of integrating emotional intelligence into language education, suggesting that a more comprehensive approach to emotional vocabulary instruction could enhance both teacher development and student learning outcomes.

## Data Availability

The original contributions presented in the study are included in the article/supplementary material, further inquiries can be directed to the corresponding author/s.
